# The Mental Disorders of Childhood

**Published:** 1908-02-15

**Authors:** G. H. Savage


### Hospital Clinics.

### THE MENTAL DISORDERS OF CHILDHOOD.

By G. H. SAVAGE, M.D., F.R.C.P.

(Abstract of a Lecture delivered on January 24, 1908, at the Medical Graduates College and Polyclinic.)

My lecture this afternoon will be limited to the true mental disorders of childhood: I shall not go into the subject of the neuroses, such as chorea, nor shall I refer directly to idiocy and similar states, though some of the cases of mental disorder approach those conditions. Many of the psychoses of infancy end in mental weakness of various kinds, but the mental diseases of this age have been very little written of. In Dr. Still's excellent Goulstonian lectures on the disorders of childhood will be found as good an account as any of the subject I have now to deal with.

First, it is quite possible for psychoses to exist before there is any real mind. Before memory begins there seems to be a big gap left which has to be filled in with a foundation of sensations and experiences. Until this gap is filled in up to the level, the building of the edifice of the mind does not properly start. Yet there are disorders, the psychoses of infancy, which may begin long before memory develops. Such disorders are met with at all ages up to puberty, and I shall deal with all those which occur in the prepubertal stage.

### The Influence of Heredity.

In most cases of juvenile psychosis there is a marked hereditary influence. This is even more noticeable in childhood than at any other age. I have already pointed out in a previous lecture that an insane mother need not bear insane children; but even when they are not insane, her children are particularly likely to show the stigmata of nervous instability. This weakness is constantly seen in the development of what I may call the most human attributes; as by defects of speech, retardation of learning to speak, retardation of assuming the erect posture, and imperfections of manual dexterity, which may be only the precursors of something worse in the offspring of insane parents. Such stigmata may be noticed even before the age of one year. Thus inexplicable attacks of screaming may be true nerve storms, and not the results of a carelessly fastened pin or an attack of indigestion. There was a child of three years old who sometimes awoke, about three hours after going to sleep, in fits of passion in which she would scratch or strike anyone who approached. The child was apparently healthy but for a slightly furred tongue and a few spots on the skin, but was of neurotic stamp and inheritance. Even after the digestive disturbances indicated had been corrected, when no exciting cause whatever could be found to exist, the attacks of passion continued as often as before. Whether pain or hallucination was the cause of the fits of rage it was difficult to ascertain.

### Results of Defective Control.

These results of defective control are not uncommon. Many of them are outgrown; but, unfortunately, others are the starting point of convulsions or of mental degradation. It is perhaps unusual to get such rages as early as the age of three, but they are not uncommon at five. They may replace, or be replaced by, fits, and some such cases have been relieved or cured by bromides. Some seven or eight years ago I was sent for to see such a child, in whom the mere fact of my arrival was sufficient to bring on a terrific nerve storm. He lay on the floor and screamed, he kicked and struggled, and nothing would induce him to behave properly. The apparent causelessness of his storms led me to suggest that he should be removed from the care of his too indulgent mother, be placed in the charge of a good nurse, be carefully and simply fed, and be given bromides. Since then he has had very few rages, and is a fine healthy boy who rides to hounds and takes his place with his fellows. But he has had occasional fits: he has been known once or twice to cry out in his sleep and to have deposited a little blood-stained saliva on his pillow. Occasional doses of bromide are still necessary to avert these attacks.

### Some Special Causes.

As to the more special causes of the psychoses of infancy and childhood, pressure on the head at birth is sometimes to blame. Injuries at birth are more likely to produce something more definite and serious, and to be associated with paralysis; but even prolonged labour may be sufficient to produce slight mental defect. In a few cases neglected ear trouble sets up morbid processes which interfere with the normal growth of the brain. In others the onset is sudden and quite obscure. Take the case of a child between two and three years old who had been up to that time perfectly healthy and normal. Without any warning he passed into a lethargic and semi-comatose state, which lasted for two or three days. The child would swallow when food was placed in its mouth. The parents were advised to prepare for convulsions or other sequelæ, but the child got gradually better. However it has never been quite the same since, and its mental development has been permanently interfered with. Among the public, teething is very popular as an explanation of mental as well as other infant troubles, but I do not myself attach much importance to it. Of local irritants which are more frequently associated with these conditions an adherent prepuce is certainly one, and it is a condition interference with which sometimes does good.

### Mental Disorders Following Specific Fevers.

Any febrile disorder in childhood may lead to mental disorder or mental weakness. Typhoid fever has been credited with especially potent powers of evil, but I have not often come across it personally. Of whooping cough, on the other hand, I have seen many instances as a cause of various psychoses: it is a common experience when inquiring into the history of mental disturbance to be told that the patient has never been the same since a bad attack of whooping cough. This disease is so bound up with the nervous system that it may be regarded as a neurosis, and I remember an interesting fact which came to light when talking to some medical men in the north of London just after a big epidemic of whooping cough in that district. One of them told me, and his experience was confirmed by others, that the disease was often found to disappear after successful vaccination, an example of how a real disease may cure a neurosis. Scarlet fever may be followed by mental disorder ranging from slight weakness to complete idiocy. I have seen a few cases of mental disease after mumps, and it is not infrequent after measles, with long complications.

Climate, too, must be considered: thus children born in India have several special dangers. First, the heat; then, malaria; thirdly, native nurses who often know only too well the uses of opium, and are occasionally guilty of initiating premature sexual abuse. It is fully recognised that there is something special in the relation of children to temperature. Some children are especially liable to rises of temperature, mostly sudden and temporary. But there are other cases of irregular pyrexia lasting some time, accompanied by restlessness, insomnia, refusal of food, chattering, laughing, and talks with the "invisible." These children do not often have terrors, but distinct hallucinatory states are not rare. Great care is required in the treatment of children during these crises. A darkened room, simple food, laxatives, a good nurse, and the absence of the parents are the chief requisites for recovery.

### Superior Polio-Myelitis.

In other cases the brain is affected much as the spinal cord is in infantile paralysis. The child is suddenly taken ill with feverishness, restlessness, and delirium, and may pass his motions and water under him: headache is sometimes, but not usually, a symptom. The cases are distinguished from meningitis by the absence of the meningeal cry and of intolerance of light. The patient may die in this condition, or may slowly recover, but even in the latter event he is no longer the child he was. The balance of the mind is completely changed, though not as a rule to the point of idiocy. Here, too, I may mention meningitis, which is sometimes recovered from: but such recovery is rarely complete, and some weak-mindedness generally remains. Inherited syphilis is often accompanied by some want of moral control, as though this too had been transmitted by heredity. Some of the most disgusting cases of child vice have been associated with inherited syphilis, and yet the patients may look healthy and be no fools intellectually. It is a question whether such mental disorders should not be regarded as allied to general paralysis of the insane: at any rate there is probably a similar diseased condition of some of the blood vessels of the brain. Probably all youthful general paralytics have congenital syphilis.

Other children seem to develop along certain lines at the expense of the rest; prodigies are in many cases examples of this. Such unequal development is a frequent occurrence in mental disorder. Sleep is often not normal, speech is often defective, mechanical aptitudes are bad, and the children when young have difficulty in feeding themselves, and have to be regularly taught to take the breast. In 1889 I saw a child five years old, the eldest of three, whose mother had been hysterical during her pregnancy. When about ten months old this infant frequently became stiff and rigid without cause, and developed passionate and destructive tendencies. He pretended to kiss his baby brother, under cover of which he bit him. As an infant this patient had seemed to lack the instinct for sucking the breast, and when five years old had still to be spoon-fed with everything. A good nurse and an open-air life cured this boy. Other children are feeble-minded from the first, and they require treatment as such. At school they learn little, and though they can talk brightly and are willing, they have not the normal power of adapting themselves naturally to their surroundings. They will carry out simple tasks, such as carting turnips on a wheelbarrow, but will be quite content to do nothing as soon as the work is finished. Yet they are not imbecile.

### Acute Mental Disorders.

I must now refer briefly to a few acute disorders of infancy. I have seen a few cases of melancholia in young children. Suicidal forms of this trouble are commoner in France than in England, but such cases do occur over here. I was once consulted about a girl aged eleven, bright, but never quite conformable to family life, though her brothers and sisters were fond of her. She was musical in a very marked degree, in fact, almost a genius; she could improvise, so I was told, and knew the principles of harmony instinctively. At ten years old she became devoutly religious, and saw angels constantly, though she was uncertain whether she heard them also; she communed, at any rate, with spirits. At the same age menstruation began, and with the onset of this she lost all faith in religion, came to the conclusion that life was not worth living, and definitely attempted to kill herself two or three times. Without having any terrific nerve storms or rages, she took the most violent likes and dislikes; and she also manifested a most precocious tendency towards the other sex.

What is to be done with such a child? To a certain extent education along musical lines might be of some avail. Unfortunately most of these children are unsociable, and are not good at games, which they in consequence dislike. In France it is not very rare to hear of suicides at ten or twelve owing to *affaires du cœur*, to the death of a canary, or some similar trivial reason.

Delirious states with pyrexia I have referred to, but actual acute mania also occurs. I once saw a child between three and four years old who had two or three times tried to injure his baby brother with a carving knife and with fire, the motive no doubt being jealousy. I asked one of the physicians of a children's hospital to admit this child temporarily while something could be arranged for him, and in the kindness of his heart the physician consented. On the first night in hospital the infant escaped into a surgical ward, where he went round with a pair of scissors and cut the bandages off nearly every child in the ward. Fortunately, perhaps, he died soon afterwards, for he was suffering from acute mania.

Hallucinations are quite common, and education has a good deal to do with this, especially religious teaching. The child's relation to the spirit world is to him a real one, and in the dark he readily visualises his conceptions of angels and so on. More children than is generally known talk to invisible hearers whose presence they imagine. It is interesting to note that hallucinations of sight are dominant in childhood, whereas in the ordinary insanity of middle age those of hearing are much the commonest. The latter do occur in children, as do those of touch also.

Masturbation is often associated with these psychoses. I have been told by patients who subsequently have become insane that they spontaneously acquired this practice in very early life, at seven years old, and even younger still.

With epilepsy there may be combined mental disorder of many different types: the medical officers of the Lingfield Colony definitely report that by reducing the number of fits they improve the mental condition.

Destructiveness, cruelty, and pyomania are common qualities in weak-minded children. Some of these are really idiots, but there are all grades between this and the very slightly defective pyomaniac, whose career is marked by a series of inexplicable conflagrations wherever he goes. Many years ago I had to give evidence about a boy of thirteen or fourteen who killed his sister, quite causelessly, with a heavy hammer, and then went out for a walk. When he returned he expressed surprise that supper was not ready, and that there was so much commotion in the house. A country life is what will be found most beneficial to all these partially weak-minded children.

